# Cancer Risk Linked to Ultra-Processed Foods: Epidemiological Findings, Potential Mechanisms, and Research Outlook

**DOI:** 10.3390/foods15213887

**Published:** 2026-10-30

**Authors:** Menglin Zhao, Xuemei Zhang

**Affiliations:** 1 School of Public Health, North China University of Science and Technology, Tangshan 063210, China; zml@ncst.edu.cn; 2 College of Life Science, North China University of Science and Technology, Tangshan 063210, China

**Keywords:** ultra-processed foods, NOVA classification, cancer risk, food processing, obesity, food additives

## Abstract

Epidemiological research on ultra-processed food (UPF) consumption and cancer has expanded rapidly; however, the evidence is uneven across cancer sites. Associations were relatively consistent for colorectal cancer, modest and partly attenuated after adjustment for breast cancer, and limited, single-cohort, or largely indirect for lung, pancreatic, renal, and prostate cancers. Recent umbrella reviews have rated the certainty of this evidence as low to very low, reflecting heterogeneous exposure classification, residual confounding, and inconsistent findings. This narrative review synthesizes the current epidemiological evidence on UPF consumption and overall and site-specific cancer risk, evaluates the biological plausibility of three mechanism clusters—obesity-driven metabolic and inflammatory pathways, processing-derived contaminants and food-contact chemicals, and food additives and their mixtures—while distinguishing human evidence from experimental models, and critically examines the NOVA classification as an exposure framework, including its application to non-Western diets. Compared with previous reviews, we appraised the evidence site by site, integrated the most recent umbrella and dose–response meta-analyses, and mapped each proposed mechanism to the tier of evidence supporting it. Standardized NOVA-based exposure assessment, repeated dietary measurements, integration of exposure biomarkers into prospective cohorts, and causal inference frameworks that distinguish confounding from mediation are prerequisites for evidence-based, UPF-specific cancer prevention recommendations.

## 1. Introduction

Cancer continues to account for a substantial share of illness and death worldwide, and its toll continues to increase as populations age and grow, urbanization proceeds, and unhealthy lifestyles spread [1,2]. Among the few available prevention levers, diet stands out. The World Cancer Research Fund and American Institute for Cancer Research have repeatedly stressed that what people eat, how much they weigh, and how active they are at the core of cancer prevention [3].

The food environment in which these dietary choices are made has changed profoundly over the past two decades. The production, marketing, and consumption of ultra-processed foods (UPFs) have increased steeply [4,5]. UPFs are industrial formulations composed largely of refined ingredients, food-derived substances, and additives, with little or no intact whole food remaining [6]. In wealthy countries, such products already supply over half of the daily energy, and in low- and middle-income countries undergoing nutritional transition, they are catching up fast [7,8]. This global dietary shift has coincided with rising rates of obesity, metabolic disorders, and other non-communicable diseases, underscoring the growing public health relevance of food processing, independent of nutrient content [9].

Early criticism of UPFs focused on their nutrient profiles: plenty of added sugars, sodium, saturated fat, and energy, but little fiber or micronutrients [10,11]. However, the health effects of UPFs appear to extend beyond nutrient composition alone [12,13]. The degree and purpose of industrial processing may introduce additional exposures, such as food additives, processing-derived contaminants, and chemicals migrating from packaging materials [14,15]. These features may, in turn, influence the gut microbiota composition, inflammatory responses, endocrine signaling, oxidative stress, and metabolic homeostasis, which offer biologically plausible pathways linking UPF consumption to carcinogenesis [16,17].

A growing body of epidemiological evidence has linked higher UPF consumption to an increased risk of obesity, cardiovascular disease, type 2 diabetes, and all-cause mortality [18,19]. Emerging prospective data suggest that UPF intake may be associated with an elevated risk of several cancers, including colorectal, breast, pancreatic, and renal cancers [20,21,22,23]. Large-scale cohort studies conducted in Europe and the United Kingdom have reported positive associations between UPF consumption and both overall cancer incidence and mortality, although the magnitude of risk varies by cancer site and population [24,25]. Meta-analyses have indicated that a higher UPF intake is associated with a modest but statistically significant increase in the overall cancer risk [26,27].

Despite these advances, important uncertainties remain. UPFs are a heterogeneous category, dietary exposure is measured imperfectly, and confounding by socioeconomic status, overall diet quality, and lifestyle is difficult to remove entirely. Disentangling the effect of processing from that of the dietary pattern remains the central methodological problem. We do not yet know how much each suspected pathway, whether obesity-related mechanisms, processing-derived contaminants, additives, endocrine-disrupting chemicals, or microbiota disruption, actually contributes to tumor development.

Against this background, this review aimed to assemble and weigh the current evidence linking UPF consumption to cancer risk. We began by summarizing the definition and classification of UPFs, followed by a critical evaluation of the epidemiological evidence for overall and site-specific cancers. We then discussed the major biological mechanisms potentially underlying UPF-associated carcinogenesis, with particular attention to obesity-related pathways, processing-derived contaminants, and food additives. Finally, we identified key methodological limitations, knowledge gaps, and priorities for future research relevant to cancer prevention and public health policy. This review differs from recent narrative reviews on the same topic in three respects: (i) we appraise the epidemiological evidence site by site, explicitly separating studies of NOVA-classified UPF exposure from indirect evidence based on dietary patterns or individual food groups; (ii) we map each proposed mechanism onto the tier of evidence supporting it, from human cohort and biomonitoring data to animal and in vitro experiments; and (iii) we critically examine the NOVA classification itself as an exposure framework, including its application to traditional foods in non-Western diets. The literature search and selection approach is described at the end of this section.

This is a narrative review. We searched PubMed/MEDLINE, Web of Science, and Scopus from database inception to August 2026 using combinations of the terms “ultra-processed food,” “NOVA,” “food processing,” “cancer,” and “neoplasm,” together with site-specific and mechanism-specific terms (e.g., “colorectal,” “breast,” “food additives,” “emulsifiers,” “acrylamide,” “endocrine disruptors”). We prioritized prospective cohort studies, systematic reviews, meta-analyses, umbrella reviews, and authoritative reports (e.g., IARC, WCRF/AICR, EFSA); case–control studies and experimental mechanistic studies were included where they provided evidence not otherwise available. Reference lists of key articles were screened for additional sources. Studies were selected to represent the strongest or most informative evidence for each topic; no formal protocol, risk-of-bias assessment, or certainty grading was applied, and the implications of this non-systematic approach are acknowledged in Section 5.

## 2. Definition of Ultra-Processed Foods

### 2.1. The NOVA Food Classification System

The NOVA food classification system was developed by Monteiro et al. to characterize foods according to the nature, extent, and purpose of industrial processing rather than nutrient composition alone. The conceptual framework was first proposed in 2009 and subsequently formalized into a four-group classification that distinguishes unprocessed or minimally processed foods, processed culinary ingredients, processed foods, and ultra-processed foods [28,29,30]. The NOVA classification framework was further operationalized through practical criteria for identifying UPFs from ingredient lists and has since become the most widely used processing-based classification system in nutritional epidemiology [6].

Under NOVA, Group 1 comprises unprocessed or minimally processed foods, including fruits, vegetables, grains, legumes, milk, eggs, and fresh meat or fish that have undergone processes such as cleaning, drying, freezing, pasteurization, and nonalcoholic fermentation. Group 2 included processed culinary ingredients derived from Group 1 foods or nature, such as oils, butter, sugar, and salt, which are primarily used for food preparation. Group 3 consisted of processed foods produced by combining Group 1 foods with Group 2 ingredients, including canned vegetables, cheese, fruits in syrup, and traditional bread. Group 4, the UPF category, comprises industrial formulations made predominantly from refined or modified food-derived substances and frequently contains additives intended to improve flavor, color, texture, stability, or palatability [6,29].

Typical UPFs include carbonated soft drinks, packaged sweet or savory snacks, confectionery, sweetened breakfast cereals, mass-produced packaged bread, instant noodles, reconstituted meat products, and ready-to-eat or ready-to-heat meals. Because processing methods are rarely disclosed on food labels, the NOVA classification relies on ingredient lists. The presence of substances rarely used in domestic kitchens, such as protein isolates, high-fructose corn syrup, hydrogenated or interesterified oils, hydrolyzed proteins, and cosmetic additives, such as flavors, colors, emulsifiers, and non-sugar sweeteners, is commonly used to identify UPFs [6].

### 2.2. Characteristics and Nutritional Profiles of UPFs

At the population level, higher UPF consumption is consistently associated with poor dietary quality. National dietary surveys indicate that UPF-rich diets exhibit higher energy density and elevated levels of free sugars, sodium, total fat, and saturated fat, concomitant with reduced dietary fiber, protein, and several micronutrients. In the United States, UPFs account for nearly 60% of the total energy intake among adults, highlighting their substantial contribution to contemporary diets [31]. Higher UPF consumption has also been prospectively associated with increased risks of being overweight, obese, and having abdominal adiposity [32]. However, the nutritional characteristics of UPFs are heterogeneous. When NOVA categories are set against nutrient profile models, the overlap proves substantial yet incomplete: most UPFs score poorly on nutrients, a few pass conventional quality criteria, and some non-UPFs score just as badly [33]. This raises the question that runs through the present review: do the observed health associations come mainly from nutrients, from processing, or from the two acting together?

Processing also reshapes the physical structure and eating properties of food. Many UPFs are soft, energy-dense, fast to eat, and hard to resist, a combination that can drive intake beyond need and blunt satiation relative to minimally processed alternatives [34]. Highly processed combinations of refined carbohydrates and fats may additionally activate reward-related neural pathways and have been hypothesized to exhibit addiction-like properties in susceptible individuals [35]. Controlled feeding trials have tested these ideas: participants on a minimally processed diet lost substantially more weight than those on a UPF-rich diet matched for presented nutrients, and experimental manipulation of texture and eating rate shifted daily energy intake in predictable directions [36,37].

UPFs may differ from minimally processed foods in terms of exposures that are not adequately characterized by conventional nutrient profiling, including food additives, thermally or processing-generated contaminants, and chemicals leaching from food-contact materials [13]. Together, these nutritional, structural, and chemical features make health effects beyond nutrient composition biologically plausible, although the contribution of each factor to carcinogenesis in humans remains an open question.

### 2.3. Challenges and Controversies in UPF Classification

Despite its widespread adoption, the NOVA framework remains subject to considerable conceptual and methodological debate. A central concern is the pronounced heterogeneity of the UPF categories. Group 4 encompasses products with markedly different nutritional compositions, ingredients, processing methods, and potential biological effects, ranging from sugar-sweetened beverages, confectionery, and processed meats to fortified whole-grain bread, breakfast cereals, flavored yogurt, and plant-based alternatives. Indeed, it is possible to construct a dietary pattern that largely meets the established dietary guidelines while deriving most of the energy from foods classified as ultra-processed [38].

This heterogeneity is also evident in prospective epidemiological studies. Adverse associations are frequently concentrated in specific UPF subgroups, particularly sugar-sweetened or artificially sweetened beverages and processed animal products, whereas some breads, breakfast cereals, and dairy products show neutral or occasionally inverse associations with cardiometabolic outcomes and multimorbidity involving cancer [39,40]. These findings suggest that associations based on total UPF intake may partly reflect the contribution of specific food categories rather than the uniform biological effect of ultra-processing. Simultaneously, associations between overall UPF intake and adverse health outcomes can persist after adjustment for nutritional quality, suggesting that nutrient composition alone may not fully explain the observed relationships [41].

The second limitation concerns the operational application of NOVA. Classification is based on the nature and purpose of processing, and this information is often unavailable in standard dietary databases and on food labels. Consequently, researchers frequently infer processing levels from product descriptions and ingredient lists, introducing potential subjectivity and misclassification [42,43]. Moreover, the categorical distinction between ultra-processed and non-ultra-processed foods may inadequately capture the continuum of processing intensity and its interactions with nutritional quality. These issues underpin the ongoing debate over whether UPF-associated health risks primarily reflect the processing itself or the nutritional and behavioral characteristics of UPF-rich dietary patterns [44,45]. Accordingly, integrated approaches combining information on processing, formulation, and nutritional quality have been proposed as complementary strategies [33,46].

These limitations have particular relevance to cancer epidemiology. Most large prospective cohorts were established before UPF research became widespread, and their dietary assessment instruments were not specifically designed to capture food-processing characteristics. Therefore, the retrospective assignment of food items to NOVA groups can introduce exposure misclassification, while differences in dietary assessment methods, food composition databases, and national food supplies further reduce comparability across studies [47]. Such methodological variation may contribute to the heterogeneity of associations observed across cancer sites and populations. Recently proposed best-practice recommendations for applying NOVA may improve classification consistency and reporting transparency [48].

Overall, NOVA provides a valuable framework for examining food processing as a dimension of dietary exposure; however, total UPF consumption should not be interpreted as a homogeneous exposure. For cancer epidemiology, future studies should complement total UPF estimates with subgroup-specific analyses, standardized classification procedures, and measures of nutritional quality and processing-related exposures. These considerations are essential when interpreting the epidemiological evidence linking UPF consumption to overall and site-specific cancer risks, which are discussed in the following section.

These challenges are amplified outside Western populations, where most UPF research to date has been conducted. Traditional breads, cheeses, and pickled or fermented foods occupy ambiguous positions within NOVA: an industrially produced flatbread or mass-market cheese may fall into Group 4 on the basis of its ingredient list, whereas a nutritionally similar artisanal product remains in Group 1 or 3; essentially, the same traditional food is classified differently according to production scale and additive content rather than its role in the diet. This ambiguity matters for cancer epidemiology in Middle Eastern and Asian settings, where such foods are dietary staples and where some traditional categories, such as salt-pickled vegetables, carry established risks that are unrelated to ultra-processing. Reflecting this geographic imbalance, the only Middle Eastern evidence included in this review comes from an Iranian case–control study and a scoping review of the regional literature (see Section 3.2.2). Prospective cohorts from non-Western populations, with culturally adapted application of NOVA, are therefore a clear research priority.

## 3. Epidemiological Evidence Linking UPF Consumption to Cancer Risk

### 3.1. Overall Cancer Incidence and Mortality

Interest in food processing as a cancer risk factor predates the NOVA classification era. The WCRF/AICR expert report judged processed meat as a convincing cause of colorectal cancer and salt-preserved foods as a probable cause of stomach cancer [3]. Notably, under the NOVA classification, these foods fall into the “processed” (Group 3) rather than “ultra-processed” (UPF, Group 4) category [6]. The WCRF/AICR report identified alcoholic beverages as convincing risk factors for various cancers [3]. These judgments are important because established dietary carcinogens are partly outside the UPF category; therefore, any UPF signal must be separated from the effects of foods already known to be harmful.

Direct evidence has begun to accumulate in prospective cohorts. In a UK analysis of 197,426 participants who completed repeated 24-h recalls (2009–2012), higher UPF consumption was associated with both greater cancer incidence and greater cancer mortality, and the association was maintained after adjustment for sociodemographic factors, smoking, alcohol consumption, BMI, physical activity, and total energy intake [25]. The strongest prospective evidence comes from the European Prospective Investigation into Cancer and Nutrition (EPIC), which enrolled 450,111 participants across 23 centers in ten European countries (1991–2001) using standardized dietary assessments [24]. Consumption of more minimally processed foods was associated with lower overall and site-specific cancer risks, whereas processed and ultra-processed food intake showed the reverse gradient; replacing only 10% of processed foods with minimally processed alternatives measurably lowered overall cancer risk [24]. These findings suggest that the UPF-cancer association persists even after adjusting for major lifestyle and metabolic confounders.

Systematic reviews and meta-analyses point in the same direction, although the certainty of their estimates has been questioned. One meta-analysis of four prospective cohorts and nine case–control studies found that higher UPF consumption was associated with increased risk of colorectal and breast cancer; compared with the lowest consumption category, participants in the highest category had an approximately 31% higher risk of colorectal cancer, an association that was significant in men but not in women [26]. A second systematic review of 11 studies (eight case–control and three prospective cohort) reported that higher UPF intake was associated with increased risks of colorectal, breast, pancreatic, and central nervous system cancers, leukemia, and overall cancer incidence; nine of the 11 studies remained positive after adjustment for major confounders, whereas evidence for prostate cancer was inconsistent [27].

Despite this growing body of evidence, the findings remain heterogeneous across populations and cancer types. The landmark NutriNet-Santé cohort first reported that each 10% increase in dietary UPF proportion was associated with a 12% higher risk of overall cancer and an 11% higher risk of breast cancer [20]. In contrast, a Spanish multicenter MCC case–control study found a significant association between UPF consumption and colorectal cancer but not with breast or prostate cancer [49]. Similarly, a multicenter case–control study of chronic lymphocytic leukemia (CLL) found no overall association with UPF intake; however, restricting analyses to cases diagnosed within one year of dietary assessment revealed a significant positive association [50], suggesting that post-diagnosis dietary changes may introduce reverse causation in retrospective studies.

Why so much heterogeneity? Exposure measurement is one reason: food-frequency questionnaires and repeated 24-h recalls do not classify UPFs equally well, so misclassification is difficult to avoid. Confounding is another reason, because socioeconomic status, smoking, alcohol consumption, physical activity, and background diet quality are entangled with UPF intake and cannot be fully adjusted. Classification itself also varies across studies: NOVA, although dominant, has been criticized for subjective criteria, internally heterogeneous food groupings, and limited attention to food-matrix properties and processing intensity [12]. These methodological limitations likely contribute to between-study heterogeneity, and ongoing efforts to refine NOVA and related classification systems should improve consistency and reproducibility in future research [12,30].

Importantly, recent syntheses have graded the certainty of the evidence base as low. The umbrella review by Lane et al. reported suggestive but low-certainty evidence linking higher UPF exposure to overall and colorectal cancer incidence and found no convincing evidence for other cancer sites, with most pooled estimates rated as low or very low quality [18]. A 2026 umbrella review of seven meta-analyses and one systematic review similarly concluded that, although higher UPF consumption was consistently associated with increased overall and colorectal cancer risk, the certainty of the evidence for cancer outcomes was low to very low, largely because of exposure misclassification, residual confounding, and heterogeneity in UPF measurements [51]. Likewise, a 2026 systematic review and dose–response meta-analysis of digestive system cancers reported positive associations overall, but effect estimates differed by study design, being stronger in case–control studies than in prospective cohorts, and the certainty of evidence was limited [52]. These syntheses do not negate the observed associations but indicate that current evidence should be regarded as consistent in direction rather than definitive in magnitude. A site-by-site summary of the epidemiological evidence is provided in Table 1. The characteristics of the principal prospective cohort studies and meta-analyses discussed in this review are summarized in Table 2.

### 3.2. Site-Specific Cancer Risks

#### 3.2.1. Colorectal Cancer

Colorectal cancer (CRC) is the third most commonly diagnosed cancer and the second leading cause of cancer-related mortality worldwide. Established risk factors include obesity and alcohol consumption, whereas a higher intake of whole grains, dietary fiber, and dairy products, together with regular physical activity, is associated with a reduced CRC risk [3]. The association between UPF consumption and CRC risk is currently supported by the most consistent epidemiological evidence among the cancer sites examined, although recent umbrella reviews have rated the certainty of this evidence as low [51,52]. A recent meta-analysis of gastrointestinal cancers found that individuals with the highest UPF intake had a significantly elevated risk of CRC, with a clear dose–response relationship [53]. Building on this evidence, large prospective cohort studies have examined the underlying mechanisms. In a study of 6155 incident CRC cases, higher UPF consumption was associated with increased CRC risk, whereas a greater intake of unprocessed or minimally processed foods was protective. Notably, this association persisted among participants with a normal BMI, suggesting that obesity does not fully account for the relationship; however, because BMI adjustment targets a downstream mediator and adiposity is incompletely measured, residual confounding cannot be excluded, and obesity independence should not be assumed [54].

Three large U.S. prospective cohorts converged on the same pattern: men with higher UPF intake had more CRC, particularly distal colon cancer, even after BMI adjustment [21]. In contrast, no significant association was observed in women. This sex disparity may be partly explained by differences in dietary patterns, as men consumed more processed meats and sugar-sweetened beverages, whereas women consumed more mixed ready-to-eat dishes and higher amounts of yogurt and dairy desserts, the latter being associated with a lower CRC risk. Hormones may supply the rest, given the suspected protective effect of estrogen and tumor-promoting potential of testosterone [21,55].

Despite this generally positive trend, not all studies concur. One large prospective analysis reported no significant association between UPF consumption and CRC risk [25]. Such discrepancies likely reflect methodological heterogeneity across studies—including variation in age range, sample size, geographic setting, and dietary assessment methodology—as well as biologically grounded sex-specific differences in colorectal tumor distribution and incidence. Women are disproportionately affected by proximal colon cancers, whereas men exhibit a higher overall incidence of colorectal cancer globally [21,24,25,55].

Collectively, the available evidence indicates that higher consumption of UPFs is associated with an elevated risk of CRC, an association that has been robustly replicated in large prospective cohort studies and pooled meta-analyses. Critically, this association persisted after multivariable adjustment for adiposity, including body mass index and waist circumference, indicating only partial mediation by obesity and implicating additional biological pathways. These pathways may be differentially modulated by sex-specific hormonal environments and dietary factors. More generally, adjustment for a downstream mediator, such as BMI, changes the causal estimand from a total effect to a direct effect and cannot exclude residual confounding by incompletely measured adiposity; persistence of an association after BMI adjustment is therefore suggestive rather than proof of obesity-independent mechanisms.

#### 3.2.2. Breast Cancer

The etiology of breast cancer is multifactorial, encompassing genetic susceptibility, family history, reproductive and hormonal determinants, environmental exposure, and modifiable lifestyle factors. A substantial proportion of breast cancer cases, particularly hormone receptor–positive subtypes, are attributable to modifiable lifestyle factors, including alcohol consumption, excess adiposity, physical inactivity, and diets low in fiber and high in UPFs [56]. Accordingly, considerable attention has been focused on the role of dietary patterns in breast cancer prevention. Epidemiological evidence consistently indicates that adherence to a Mediterranean dietary pattern, characterized by a high intake of fruits, vegetables, legumes, whole grains, and unsaturated fats, is associated with reduced breast cancer risk, whereas Western dietary patterns rich in processed meats, refined grains, and highly processed foods are associated with an increased breast cancer risk [57,58,59].

Whether UPFs add anything beyond these broad patterns remains unclear. NutriNet-Sante again led the way: a 10% higher UPF share of the diet came with roughly 11% higher breast cancer risk, hinting that processing itself might matter [20], a suggestion echoed by studies linking energy-dense and fast-food-type patterns to breast cancer [60]. However, not all studies support this association. Case–control studies conducted in Spain and Iran found no significant relationship between overall UPF consumption and breast cancer risk [49,61]. Notably, the MCC-Spain study observed a significant association in unadjusted analyses that became non-significant after controlling for total energy intake and alcohol consumption. These findings suggest that the apparent UPF-breast cancer association may partly reflect confounding by established breast cancer risk factors, such as overall dietary quality, excess energy intake, and alcohol consumption.

Despite these inconsistencies at the individual study level, pooled evidence from systematic reviews and meta-analyses provides stronger support for a modest positive association. A meta-analysis of six observational studies (462,292 participants) demonstrated that individuals with the highest UPF consumption had a higher risk of breast cancer than those with the lowest intake, with each 10% increase in dietary UPF proportion associated with an approximately 5% higher risk, indicating a linear dose–response relationship [62]. Another systematic review reported a 25% higher risk of breast cancer among high versus low UPF consumers [63]. These findings indicate a modest but consistent positive association between UPF intake and the risk of breast cancer.

#### 3.2.3. Lung Cancer

Lung cancer remains the leading cause of cancer-related mortality worldwide. Although smoking is the primary risk factor, an increasing proportion of cases occur among never-smokers, suggesting that other environmental and lifestyle factors, including diet, contribute to disease risk [64,65]. In this context, UPF-rich dietary patterns have been discussed as potential modifiable contributors to lung cancer risk, although direct UPF-specific evidence remains scarce.

It should be emphasized that direct evidence based on NOVA-classified UPF exposure is lacking for lung cancer; the studies described below largely assess Western dietary patterns or individual food groups that are typical components of UPF-rich diets and, therefore, provide only indirect evidence. A large U.S. case–control study found that Western dietary patterns, characterized by high consumption of processed meats, refined grains, high-fat dairy, and sugary foods, were associated with increased lung cancer risk, whereas prudent diets rich in fruits, vegetables, and whole grains were protective [66]. In the UK Biobank (416,588 participants), higher red and processed meat intakes were linked to increased lung cancer incidence, whereas fish, whole grains, fruits, vegetables, and minimally processed foods reduced the risk in the opposite direction [67]. A Chinese case–control study yielded consistent results, indicating that adherence to Western dietary patterns was significantly associated with an elevated risk of lung cancer, whereas greater consumption of traditional plant-based diets was associated with a reduced risk [68].

However, the evidence remains inconclusive. For example, the EPIC study found no significant association between red or processed meat intake and lung cancer risk [69]. These discrepancies likely stem from variations in dietary assessment methodologies, study populations, and exposure definitions. Most existing studies have assessed broad dietary patterns or isolated food groups, not UPFs as rigorously classified under the NOVA framework, thereby compromising cross-study comparability and impeding causal attribution specifically to UPFs. A key analytical limitation is the substantial confounding effect of smoking, which exhibits strong positive associations with both unhealthy dietary behaviors and the risk of lung cancer. Although most studies adjusted for smoking status, residual confounding may have persisted. Interestingly, dietary effects may be more detectable among never-smokers, whose lower baseline risk may make the contribution of other exposures, such as UPFs, more apparent [64,65].

Heterogeneity in lung cancer histology may further complicate interpretation, as few studies have examined whether UPF-cancer associations differ across histological subtypes or whether they are modified by smoking status, sex, or genetic susceptibility, leaving key questions about vulnerable subgroups unanswered. Future large-scale prospective studies using a standardized NOVA classification, repeated dietary assessments, and analyses stratified by smoking status and histological subtype are needed to clarify the role of UPFs in lung carcinogenesis.

#### 3.2.4. Pancreatic Cancer

Pancreatic cancer is among the most aggressive malignancies, with an overall five-year survival rate of less than 15% owing to delayed diagnosis and rapid clinical progression. Established risk factors include cigarette smoking, obesity, type 2 diabetes mellitus, chronic pancreatitis, and genetic susceptibility, along with a growing body of evidence implicating dietary habits in pancreatic carcinogenesis [70,71]. Given the strong link between UPF consumption and excess adiposity, recent studies have focused on whether UPFs modulate pancreatic cancer risk independently.

A systematic review and meta-analysis reported that obesity is associated with a 28% increase in pancreatic cancer mortality [70]. Similarly, another meta-analysis demonstrated that each 1 kg/m^2^ increase in pre-diagnostic body mass index (BMI) was correlated with a 10% increase in pancreatic cancer mortality, underscoring excess adiposity as a key determinant of disease prognosis [71]. Because high UPF intake is consistently linked to weight gain and metabolic dysfunction, these findings lend biological plausibility to the dietary role in pancreatic cancer, although they do not demonstrate direct causality.

To date, direct epidemiological evidence linking UPF consumption to pancreatic cancer is limited. The most compelling prospective evidence comes from the Prostate, Lung, Colorectal, and Ovarian (PLCO) Cancer Screening Trial, which investigated UPF consumption and pancreatic cancer risk in U.S. adults. During follow-up, a higher UPF intake was significantly associated with an elevated risk of pancreatic cancer. Notably, dose–response analyses revealed a nonlinear positive relationship, suggesting that the risk increases disproportionately at high exposure levels. This association persisted after adjusting for demographics, smoking, BMI, diabetes, total energy intake, and overall diet quality, indicating that the observed risk was not fully explained by these established risk factors [22]. It should be emphasized that this direct evidence derives from a single cohort, and replication in independent populations is required before the association can be considered established.

In contrast, a population-based case–control study involving 307 cases and 322 controls observed a positive association between bread consumption and pancreatic cancer risk [72]; however, because bread encompasses diverse products spanning minimal to ultra-processing, these results cannot be directly generalized to UPFs. Similarly, the findings regarding red and processed meats remain equivocal [73]. While a positive association between red meat intake and pancreatic cancer was observed across two prospective cohorts, prospective analyses from the European Prospective Investigation into Cancer and Nutrition (EPIC) and the Cancer Prevention Study-II Nutrition Cohort found no significant relationship [74,75]. These discrepancies highlight the limitations of single-food group analyses, which fail to capture the complex and cumulative exposure represented by the total UPF intake.

#### 3.2.5. Renal Cell Carcinoma

Renal cell carcinoma (RCC) accounts for the majority of kidney cancer cases and represents a growing public health challenge worldwide. Although its molecular pathogenesis is multifaceted, accumulating evidence underscores the pivotal role of modifiable lifestyle factors, including obesity, hypertension, smoking, and poor dietary quality, in disease development and progression [76,77]. Given the robust link between UPF consumption, weight gain, and cardiometabolic dysfunction, increasing attention has been directed toward the potential contribution of UPFs to renal carcinogenesis.

To date, direct epidemiological evidence evaluating the association between UPF consumption and RCC risk has been limited. The primary prospective evidence was derived from a large investigation by Li et al. involving 101,668 U.S. adults, which evaluated the association between UPF intake, incident RCC, and disease-specific mortality [23]. Over the follow-up period, higher UPF consumption was significantly associated with elevated risks of both incident RCC and RCC mortality in a linear, dose-dependent manner. Notably, these risk increases were more pronounced among participants younger than 65 years, suggesting that age may modulate biological susceptibility to long-term exposure to UPFs in this population. These associations persisted after adjusting for major potential confounders, including demographic traits, lifestyle behaviors, body mass index (BMI), and total caloric intake, indicating an effect independent of established risk factors [23].

These direct cohort findings are indirectly supported by broader evidence linking poor dietary quality to RCC. A review of observational studies identified diet quality as an important modifiable determinant of RCC risk, showing that adherence to healthy dietary patterns consistently confers protection [76]. Similarly, a prospective cohort study of postmenopausal women linked higher dietary quality to reduced kidney cancer-specific and all-cause mortality [77]. Although these broader studies did not explicitly evaluate UPFs using standardized processing frameworks, they reinforced the general influence of diet on renal carcinogenesis.

However, the evidence regarding specific UPF subcategories is equivocal. For instance, sugar-sweetened beverages (SSBs) have been frequently investigated because of their adverse effects on metabolism and health. However, in the European Prospective Investigation into Cancer and Nutrition (EPIC) study, soft drink consumption was not significantly associated with RCC incidence after controlling for BMI, indicating that excess adiposity may mediate much of the observed relationship between individual sugary beverages [78]. This contrast emphasizes the importance of assessing total complex UPF exposure rather than relying solely on single food items, which may fail to capture the synergistic effects of ultra-processed food. Overall, the RCC evidence is encouraging but rests largely on one prospective cohort, and these findings should be regarded as preliminary until they are replicated in other populations.

#### 3.2.6. Prostate Cancer

Prostate cancer is the second most frequently diagnosed malignancy among men globally and remains a leading cause of cancer-related mortality. Established risk factors include advancing age, family history, genetic susceptibility, and African ancestry, whereas the causal contribution of dietary factors remains unclear. In recent years, attention has increasingly focused on whether UPF consumption promotes prostate carcinogenesis through mechanisms involving systemic metabolic dysfunction, hyperinsulinemia, and inflammation. However, compared with malignancies such as colorectal or breast cancer, direct epidemiological evidence linking UPF intake to prostate cancer incidence is scarce.

Current evidence is predominantly indirect, relying on studies that examine the biological intermediates or specific nutritional components associated with UPF consumption. For example, several studies have demonstrated that higher UPF intake correlates with elevated circulating levels of systemic inflammatory markers, including C-reactive protein (CRP) [17,79]. Systemic low-grade inflammation and metabolic syndrome are well-documented contributors to the initiation and aggressive progression of prostate tumors [80,81]. Although these associations provide strong biological plausibility, they do not offer direct epidemiological confirmation of UPF-driven prostate carcinogenesis.

Similarly, the individual nutritional components abundant in UPFs have been independently evaluated. Industrially produced trans-fatty acids (TFAs) have been scrutinized because of their capacity to induce cellular oxidative stress and systemic inflammation. A systematic review and meta-analysis observed that higher total TFA intake was associated with an increased risk of prostate cancer, although the risk estimates varied according to the specific fatty acid isomers [82]. Supporting this, a multiethnic case–control study found positive associations between TFA exposure and prostate cancer in African American, European American, and Ghanaian men, suggesting a consistent relationship across diverse ancestral backgrounds [83]. Nevertheless, these studies evaluated single isolated nutrients rather than a holistic UPF-processing matrix.

Dietary nitrates and nitrites, frequently added as preservatives to processed meat, represent another group of implicated compounds. In a large prospective cohort study in the U.S., higher dietary nitrite intake was significantly associated with an increased risk of advanced prostate cancer, whereas no clear association was observed with fatal prostate cancer [84]. These findings suggest that specific dietary exposures may differentially affect disease aggressiveness at different clinical stages. However, as nitrates and nitrites originate from both synthetic processing additives and natural plant sources, these observations cannot be interpreted as direct evidence of UPF-induced harm in humans.

Another emerging concern involves endocrine-disrupting chemicals (EDCs) that can migrate into food products from synthetic packaging materials or processing machinery. Preclinical experimental models indicate that these xenobiotics can disrupt androgen receptor signaling and promote prostate cell proliferation [85]. However, epidemiological data directly linking UPF intake to altered internal EDC exposure levels and subsequent prostate cancer incidence remain sparse, leaving this hypothesized pathway largely unconfirmed in clinical settings.

## 4. Potential Biological Mechanisms Underlying UPF-Associated Carcinogenesis

**Figure 1:**
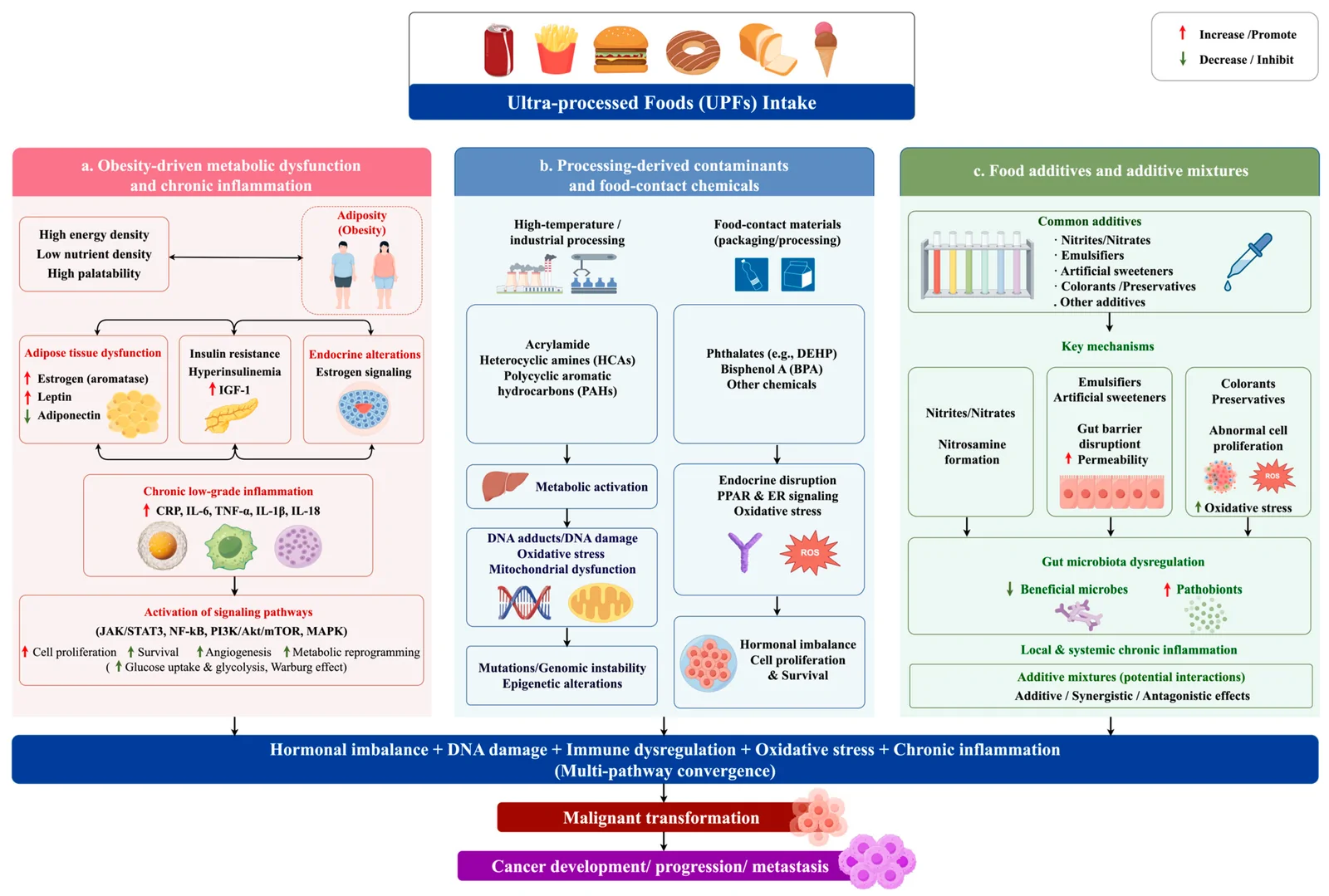
Potential biological mechanisms linking UPF consumption to carcinogenesis. UPFs may contribute to carcinogenesis through three interconnected pathways: (**a**) obesity-driven metabolic dysfunction and chronic inflammation; (**b**) exposure to processing-derived contaminants and food-contact chemicals, resulting in genotoxicity, oxidative stress, and endocrine disruption; and (**c**) exposure to food additives and additive mixtures, potentially affecting nitrosamine formation, gut barrier integrity, microbiota composition, and inflammatory responses. These pathways may converge on hormonal imbalance, DNA damage, immune dysregulation, oxidative stress, and chronic inflammation, thereby promoting malignant transformation and cancer progression.

The mechanisms reviewed below differ markedly in the tier of evidence supporting them. Obesity-driven pathways are supported by controlled human feeding trials and prospective epidemiology, whereas the contaminant and additive pathways rest largely on hazard characterization, experimental animal, and in vitro studies, and a small but growing number of human biomonitoring and cohort studies. Throughout this section, we indicate the evidentiary tier on which each proposed mechanism currently rests, and we emphasize that biological plausibility demonstrated in experimental systems does not by itself establish carcinogenic effects at human dietary exposure levels. The three proposed mechanism clusters and their convergence on tumor-promoting processes are illustrated in Figure 1.

### 4.1. Obesity-Driven Metabolic Dysfunction and Chronic Inflammation

Obesity is a well-established mechanism through which UPF intake may contribute to cancer development. Epidemiological studies have consistently demonstrated that diets high in UPFs exhibit inferior overall nutritional quality compared to those based predominantly on unprocessed or minimally processed foods [11,86,87]. Such dietary patterns are typically characterized by higher energy and increased intakes of free sugars, saturated fats, trans-fatty acids, and sodium, alongside reduced intakes of dietary fiber, protein, potassium, and other micronutrients [11,86,87]. Notably, findings from a randomized controlled feeding trial indicated that participants assigned to an ultra-processed diet consumed substantially more energy and gained greater weight than those on an unprocessed diet [88]. Thus, excess energy intake and the resulting adiposity constitute crucial biological links between UPF consumption and carcinogenesis.

Adipose tissue functions not only as an energy-storage organ but also as an active endocrine and immunological compartment capable of modulating the pro-tumorigenic systemic microenvironment [89]. Excess adiposity induces adipocyte hypertrophy, tissue hypoxia, immune cell infiltration, extracellular matrix remodeling, and altered adipokine secretion, converging to drive chronic low-grade inflammation and metabolic dysfunction [89,90]. In obesity, increased recruitment and activation of macrophages in adipose tissue upregulates the production of pro-inflammatory mediators, including tumor necrosis factor-α (TNF-α), interleukin-6 (IL-6), and IL-1β [90,91]. These mediators activate oncogenic signaling pathways, such as NF-κB, JAK/STAT3, and MAPK, thereby promoting cell survival, proliferation, angiogenesis, and apoptosis resistance. Obesity-associated inflammasome activation further amplifies this inflammatory milieu via the enhanced production of IL-1β and IL-18 [90,91].

The consequences of obesity-associated inflammation may differ according to tumor type and tissue context [92,93]. In colorectal cancer, for example, obesity-associated inflammatory signaling, including IL-1β-related pathways, may promote intestinal epithelial proliferation and create a microenvironment conducive to tumor development [91]. In pancreatic cancer, the interplay between dysfunctional adipose tissue and the tumor microenvironment may facilitate inflammatory cell recruitment, metabolic remodeling, and tumor progression [94]. These observations offer a potential explanation for why obesity-associated cancers may be particularly susceptible to the metabolic effects of sustained UPF consumption.

Obesity modulates systemic endocrine signaling. Adipose tissue expresses aromatase, which converts androgens into estrogens; consequently, increased adiposity can elevate estrogen exposure, particularly after menopause [95]. Estrogen signaling through estrogen receptors can activate proliferative pathways, such as PI3K/Akt and MAPK signaling, thereby providing an important mechanistic link between excess adiposity and hormone-sensitive malignancies [95,96]. This pathway may be particularly relevant to breast and endometrial cancers, although the biological effects of estrogen signaling are strongly dependent on the tissue and tumor context.

A second major metabolic link involves insulin resistance and the insulin/IGF axis. Obesity-associated insulin resistance elicits compensatory hyperinsulinemia, which has been implicated both epidemiologically and mechanistically in several obesity-related cancers [97,98]. Hyperinsulinemia reduces circulating IGF-binding proteins, particularly IGFBP-1 and IGFBP-2, consequently elevating IGF bioavailability [98]. Activation of insulin and IGF-1 receptor signaling then stimulates downstream PI3K/Akt/mTOR and Ras/Raf/MAPK pathways, which promote cellular proliferation, survival, and anabolic metabolism. These pathways also facilitate metabolic reprogramming toward increased glucose uptake and glycolysis, thereby meeting the high biosynthetic and energetic demands of proliferating tumor cells [97,98,99,100]. Thus, sustained metabolic dysfunction induced by excess adiposity creates a milieu conducive to both tumor initiation and progression.

Altered adipokine secretion is an additional mechanistic link between adiposity and carcinogenesis. Obesity is characterized by increased leptin signaling and reduced adiponectin availability. Leptin promotes proliferative, inflammatory, invasive, and epithelial–mesenchymal transition programs through the JAK/STAT, MAPK, and PI3K/Akt/mTOR signaling pathways [101,102,103]. Conversely, adiponectin exerts anti-inflammatory and growth-inhibitory effects, partly through the activation of AMP-activated protein kinase (AMPK) and suppression of mTOR-dependent signaling [104]. The obesity-associated shift toward a high leptin/low adiponectin state fosters a pro-tumorigenic metabolic and inflammatory milieu.

Adipose tissue expansion induces local hypoxia and cellular stress, further exacerbating inflammation and metabolic dysregulation [105]. Hypoxia-inducible signaling can stimulate angiogenesis and glycolytic adaptation, processes that may support tumor growth once malignant transformation has occurred [106]. Emerging evidence suggests that some obesity-associated molecular alterations may persist even after weight loss. Persistent changes in DNA methylation, histone modifications, and other epigenetic regulatory processes have therefore been proposed as a potential form of “metabolic memory” that could extend the carcinogenic consequences of previous obesity [107], although the relevance of this phenomenon to UPF-associated cancer risk requires further investigation.

Obesity is a major biological pathway linking UPF consumption and cancer. Excess adiposity promotes metabolic, endocrine, and inflammatory dysregulation, collectively creating a systemic environment conducive to tumorigenesis in the body. However, obesity alone is unlikely to fully explain the UPF-associated cancer risk, highlighting the potential contribution of obesity-independent mechanisms related to food processing and formulation.

### 4.2. Food Processing-Derived Contaminants and Food-Contact Chemicals

Food processing and packaging introduce potentially carcinogenic chemicals, representing an additional mechanism linking UPF consumption to cancer. High-temperature processing, including frying, baking, roasting, and grilling, generates contaminants such as acrylamide, heterocyclic amines (HCAs), and polycyclic aromatic hydrocarbons (PAHs). Additionally, extensive industrial processing and packaging increase exposure to food-contact chemicals, including phthalates and bisphenols. These compounds promote carcinogenesis through genotoxicity, oxidative stress, endocrine disruption and dysregulated cellular signaling.

Acrylamide is primarily generated via the Maillard reaction between asparagine and reducing sugars during the thermal processing of carbohydrate-rich foods. Following ingestion, acrylamide is metabolized to glycidamide, a reactive metabolite that forms DNA adducts and elicits genotoxic damage. Experimental studies further demonstrate that acrylamide exposure induces oxidative stress and DNA damage [108,109]. However, although acrylamide is carcinogenic in experimental models, evidence linking dietary exposure at typical human intake levels to cancer remains inconclusive.

Other heat-generated contaminants may also contribute to comparable genotoxic effects. HCAs, including PhIP and MeIQx, are primarily formed during the high-temperature cooking of meat. Following metabolic activation, reactive HCA metabolites form DNA adducts and induce mutations in cancer-related genes, leading to tumorigenesis. PAHs, generated during incomplete combustion and certain high-temperature cooking processes such as grilling and smoking, can undergo cytochrome P450-mediated metabolic activation to form DNA-reactive metabolites. Persistent DNA adduct formation and subsequent mutagenesis provide plausible mechanisms linking these compounds to carcinogenesis [110]. However, HCAs and PAHs are not specific to UPFs; their exposure is strongly influenced by food type and cooking method, making it difficult to establish their independent contributions to the risk of UPF-associated cancer.

Food-contact chemicals represent another potential exposure pathway. Biomonitoring data from the U.S. National Health and Nutrition Examination Survey showed that higher UPF consumption was associated with increased urinary concentrations of several phthalate metabolites, although no associations were observed for total DEHP metabolites or bisphenols [14]. These findings suggest that UPF consumption may serve as a marker for increased exposure to certain chemicals used during food production, processing, and packaging. Phthalates, such as di(2-ethylhexyl) phthalate (DEHP) and its metabolites, can interfere with nuclear receptor signaling and have been implicated in oxidative stress, metabolic disturbances, and endocrine dysfunction [111,112]. Bisphenol A (BPA), another widely studied endocrine-disrupting chemical, interacts with estrogen and other nuclear or membrane receptors and modulates signaling pathways and the tumor microenvironment, providing biological plausibility for its involvement in hormone-sensitive carcinogenesis [112,113].

In summary, processing-derived contaminants and food-contact chemicals provide plausible obesity-independent mechanisms linking UPFs to cancer. However, because these exposures are neither unique to UPFs nor consistently correlated with UPF intake, their contributions to the observed epidemiological associations remain unknown. Studies integrating UPF assessment with biomarkers of chemical exposure are needed to elucidate these relationships.

### 4.3. Food Additives and Additive Mixtures

Food additives represent an additional mechanism linking UPF consumption to cancer. UPFs typically contain preservatives, colorants, sweeteners, emulsifiers, and other additives that enhance shelf life, texture, appearance, and palatability. Although authorized food additives are evaluated under established regulatory frameworks, concerns persist regarding chronic exposure, combined exposure to multiple additives, and potential effects that are not adequately captured by conventional single-compound assessments [114,115,116].

Nitrites and nitrates are extensively employed as preservatives and color stabilizers in processed meats. Under specific physiological conditions, nitrites contribute to the endogenous formation of N-nitroso compounds (NOCs), including carcinogenic nitrosamines [117]. Upon metabolic activation, certain NOCs produce reactive intermediates that alkylate DNA and generate mutagenic lesions, such as O^6^-methylguanine, thereby promoting carcinogenesis [118]. Epidemiological studies further indicate that elevated dietary exposure to nitrites and NOCs is associated with increased risks of certain cancers, although these associations vary by dietary source and cancer site [119].

Artificial sweeteners have attracted considerable attention, although their carcinogenic effects are controversial. Aspartame is one of the most extensively studied artificial sweeteners. In 2023, the International Agency for Research on Cancer classified aspartame as “possibly carcinogenic to humans” (Group 2B), based on limited evidence of carcinogenicity in humans, while the Joint FAO/WHO Expert Committee on Food Additives maintained the acceptable daily intake at 0–40 mg/kg body weight [114]. Thus, current evidence does not establish a causal relationship between aspartame consumption within the recommended exposure levels and cancer; however, continued investigation of long-term and high-level exposure remains warranted [120,121]. Notably, prospective human evidence has begun to complement these hazard assessments: in the NutriNet-Santé cohort (102,865 adults), higher overall consumption of artificial sweeteners was associated with increased cancer risk (hazard ratio [HR] 1.13, 95% CI 1.03–1.25), and aspartame intake was associated with higher overall cancer risk (HR 1.15, 95% CI 1.03–1.28) and breast cancer risk specifically [122]. Although observational and subject to residual confounding, these findings extend the evidence base for additives beyond the regulatory hazard classification. Proposed mechanisms, including oxidative stress, metabolic alterations, and endocrine-related effects, have not been sufficiently established in humans yet.

Titanium dioxide (TiO_2_; E171) exemplifies the uncertainty surrounding previously authorized food additives. Concerns regarding genotoxicity led the European Food Safety Authority to conclude in 2021 that E171 can no longer be considered safe as a food additive. Experimental evidence suggests that ingested TiO_2_ particles may interact with the intestinal environment, induce oxidative stress and inflammatory responses, and potentially compromise epithelial barrier integrity [115]. However, direct evidence demonstrating that dietary E171 exposure increases the risk of human cancer remains insufficient, and mechanistic findings from experimental models should not be directly extrapolated to human carcinogenesis.

Emulsifiers are an emerging concern. Experimental studies have demonstrated that widely used emulsifiers, particularly carboxymethylcellulose (CMC) and polysorbate 80 (P80), alter gut microbial composition, compromise the mucus–microbiota interface, and induce low-grade intestinal inflammation [15,16,123]. These alterations enhance microbial contact with the intestinal epithelium and activate innate immune and inflammatory signaling pathways, including TLR- and NF-κB-related pathways. These microbiota–barrier–inflammation interactions establish a biologically plausible connection between chronic emulsifier exposure and colorectal carcinogenesis. However, most mechanistic evidence remains confined to animal and experimental models, and the translational relevance of typical human dietary exposure requires further investigation. Prospective human evidence has also begun to emerge. In the NutriNet-Santé cohort (approximately 92,000 adults), higher carrageenan intake was associated with an increased breast cancer risk (HR 1.32), whereas no emulsifier was significantly associated with colorectal cancer risk [124]. The absence of a colorectal signal in humans, despite strong experimental support for the microbiota–barrier–inflammation axis, illustrates the gap between mechanistic plausibility and population evidence that future cohort studies with additive-specific exposure assessments must close.

An emerging concern is that UPFs typically contain mixtures of additives, rather than individual compounds. Chronic co-exposure may result in additive, synergistic, or antagonistic biological effects that cannot be predicted from conventional single additive safety assessments. Recent epidemiological studies evaluating additive mixtures offer an important framework for addressing this issue; however, the evidence remains in its early stages. Future studies integrating mixture-based exposure assessment, biomarkers, and prospective cancer outcomes will therefore be critical for determining whether combinations of food additives contribute to UPF-associated carcinogenesis.

## 5. Limitations and Future Perspectives

Stepping back, the epidemiology tells a consistent directional story (more UPFs, more cancer), but one whose magnitude, and occasionally, whose existence, varies across cancer sites, populations, and study designs, such that causality cannot yet be claimed. Several methodological and conceptual challenges must be addressed before UPF-specific cancer prevention recommendations can be made.

First, the exposure itself. NOVA dominates the field, yet its Group 4 sweeps together products with markedly different compositions and plausible effects: sugar-sweetened beverages and processed meats on one side, and fortified cereals, packaged whole-grain breads, and certain plant-based products on the other. Because NOVA characterizes processing rather than nutrients, foods with substantially different nutritional profiles share a category, and applying the system to dietary datasets involves subjective judgment that varies between studies. Future work should improve the standardization and reproducibility of UPF classification and, where appropriate, integrate processing characteristics with nutritional quality and food-subgroup information, so that effects attributable to processing itself can be distinguished from those arising from the nutrient content of particular UPFs.

The second major challenge lies in the complex interplay between UPF consumption, adiposity, dietary quality, and socioeconomic and lifestyle factors. Obesity functions as both a mediator of UPF-associated cancer risk and an independent carcinogenic factor. UPF-rich diets promote excess energy intake and weight gain, whereas obesity subsequently contributes to carcinogenesis via metabolic, endocrine, and inflammatory pathways. Nevertheless, associations with certain cancers persisted after adjusting for body mass index, indicating that adiposity alone does not fully account for the observed relationships. Similarly, high UPF consumption is associated with poor dietary quality, including reduced fiber and micronutrient intakes and elevated consumption of added sugars, saturated fats, and sodium [125]. However, whether UPFs exert adverse effects independent of their nutritional composition remains unclear. Interpreting BMI-adjusted estimates also requires caution because adiposity lies on the causal pathway between UPF consumption and cancer. Adjustment for BMI changes the estimand from a total effect to a direct effect and may introduce over-adjustment or collider bias. Moreover, BMI is an incomplete measure of adiposity; therefore, residual confounding remains possible.

Socioeconomic status and related lifestyle characteristics further complicate causal interpretation. UPF consumption is socially patterned and clustered, with differences in food accessibility, education, physical activity, smoking, alcohol consumption, and other health behaviors [19,126]. Conventional multivariable adjustments may not fully eliminate these interrelated sources of confounding. Differences in study design are also consequential, with stronger associations frequently observed in case–control studies than in prospective cohorts, potentially reflecting recall and selection biases, whereas adjustment for energy intake, alcohol consumption, and other dietary factors can substantially attenuate some associations [46]. These issues underscore the necessity of distinguishing more carefully among confounders, mediators, and effect modifiers, rather than treating all covariates simply as adjustment variables.

Future epidemiological studies should prioritize large, diverse, long-term prospective cohorts with repeated dietary assessments. Repeated measurements would better capture changes in UPF consumption over time and reduce exposure misclassification, which is particularly important given the long latency period of cancer development. Analyses should examine both total UPF intake and specific UPF subcategories, while stratification by sex, age, socioeconomic background, lifestyle characteristics, genetic susceptibility, and tumor subtype may help identify vulnerable populations. Advanced causal inference approaches, including mediation analysis, substitution models, and, where appropriate, instrumental variable methods, could further clarify the relative contributions of adiposity, dietary quality, and other intermediate pathways. The observation that UPF consumption may be associated with metabolic liver disease without a corresponding clear association with hepatocellular carcinoma also highlights the importance of considering disease stage and intermediate metabolic phenotypes when interpreting cancer outcomes [127].

Greater attention should be directed toward identifying specific processing-related exposures that may underlie epidemiological associations. Current studies rarely integrate UPF intake with biomarkers of exposure to processing-generated contaminants, food additives, and food-contact chemicals. Combining detailed dietary assessments with metabolomics, exposomics, microbiome profiling, and chemical biomarkers could help determine whether the observed associations arise primarily from nutritional deterioration, excess energy intake, processing-related chemicals, or combinations of these factors. Particular attention should be paid to chronic exposure to nitrates, nitrites, artificial sweeteners, emulsifiers, and other additives, as well as structural changes in the food matrix induced by industrial processing.

As discussed in Section 4.3, the potential additive, synergistic, or antagonistic effects of additive mixtures remain an important but underexplored area of research. Experimental models can characterize biologically plausible interactions among additives and contaminants, whereas prospective epidemiological studies incorporating mixture analysis methods and exposure biomarkers can evaluate their relevance at realistic dietary exposure levels. Short-term controlled feeding studies and substitution analyses may provide complementary evidence by determining whether replacing specific UPF categories with minimally processed alternatives improves the intermediate metabolic, inflammatory, and molecular markers associated with cancer development.

Beyond individual health outcomes, the broader implications of UPFs for food-system sustainability warrant further investigation. The production, packaging, transportation, and marketing of highly processed foods may have environmental consequences that are not captured by conventional nutritional assessments. Integrating human health outcomes with environmental indicators could therefore provide a more comprehensive framework for evaluating the role of UPFs within sustainable food systems.

Finally, the limitations of this review itself should be acknowledged. This is a narrative review: although we searched major databases and prioritized prospective and pooled evidence (see Section 1), study selection was not systematic, and no formal risk-of-bias or certainty assessment was performed; therefore, relevant studies may have been missed. We restricted the search to English-language publications, which may under-represent evidence from regions where UPF research is published in other languages, and publication bias toward positive findings may distort the literature summarized here. Our appraisal should therefore be read as an interpretive synthesis rather than an exhaustive evaluation of the evidence.

Overall, future research should move beyond the question of whether total UPF consumption is associated with cancer and toward determining which UPFs, processing-related exposures, and populations drive the observed associations. Standardized exposure classification, repeated dietary assessment, improved causal-inference methods, and integration of epidemiological data with mechanistic and biomarker evidence will be essential for strengthening causal interpretation. Until such evidence becomes available, public health recommendations should emphasize established principles of healthy dietary patterns, while avoiding overly broad assumptions that all foods classified as UPFs confer comparable cancer risk.

## Figures and Tables

**Table 1:** Summary of epidemiological evidence linking UPF consumption to site-specific cancer risk.

Cancer Type	Strength and Consistency of Evidence	Main Epidemiological Findings	Key Limitations	Research Priorities
Colorectal cancer	Relatively consistent; supported by multiple prospective cohorts and meta-analyses, but certainty rated low by recent umbrella reviews	Higher UPF intake is associated with increased incidence of colorectal cancer in most prospective cohorts and meta-analyses, with dose–response patterns and stronger associations in men.	Sex differences and heterogeneity across UPF subcategories remain unexplained, and evidence is concentrated in European and North American populations.	Clarify sex-specific mechanisms and obesity-independent pathways using standardized UPF classification
Breast cancer	Moderate; modest pooled associations, with mixed findings among individual studies	Pooled analyses suggest a modest increase in breast cancer incidence; in several individual studies, associations were attenuated after adjustment for energy intake and alcohol consumption.	Substantial heterogeneity and residual confounding limit causal inference, and subtype-specific evidence is scarce.	Subtype-specific prospective studies with repeated dietary assessments should be conducted.
Lung cancer	Limited and indirect; based mainly on dietary-pattern or food-group studies rather than NOVA-classified UPF exposure	Western dietary patterns and processed meat intake have been associated with a higher lung cancer incidence in some, but not all, cohorts; however, direct evidence on total UPF intake is lacking.	Residual confounding by smoking, reliance on indirect exposure proxies, and few subtype-specific analyses.	Evaluate NOVA-classified UPF exposure in never-smokers and across histological subtypes
Pancreatic cancer	Limited; direct evidence derives largely from a single prospective cohort (PLCO)	One large cohort reported a positive, non-linear association between UPF intake and pancreatic cancer incidence that was robust after adjustment for BMI, diabetes, and diet quality.	Single-cohort evidence without replication; obesity may act as both a mediator and confounder.	The findings should be validated in independent prospective cohorts, and obesity-independent mechanisms should be investigated.
Renal cell carcinoma	Limited; direct evidence derives largely from a single prospective cohort	Higher UPF intake was associated with increased renal cell carcinoma incidence and cancer-specific mortality in a large cohort, with linear dose–response patterns and stronger associations in participants younger than 65 years.	Single-cohort evidence; findings require replication in diverse populations.	Replicate in diverse populations and clarify the underlying metabolic mechanisms
Prostate cancer	Insufficient; mostly indirect evidence	Direct evidence is scarce, and most studies examine dietary components related to UPFs rather than total UPF intake.	Limited prospective evidence and substantial biological heterogeneity	Perform large prospective studies integrating biomarkers and molecular subtypes

Note: Evidence ratings reflect the authors’ qualitative appraisal of the number, design, and consistency of studies using NOVA-classified UPF exposure, informed by recent umbrella reviews that graded the certainty of evidence as low to very low [18,51,52]; they are not formal GRADE ratings. Cancer incidence and mortality are distinguished as reported.

**Table 2:** Characteristics of key prospective cohort studies and meta-analyses on UPF consumption and cancer risk.

Study or Review [Ref.]	Design and Population	UPF Measure and Outcome	Main Findings	Key Limitations
Fiolet et al., 2018 (NutriNet-Santé) [20]	Prospective cohort; 104,980 French adults	UPF as % of diet by weight (repeated 24-h recalls); overall and breast cancer incidence	Each 10% increment in dietary UPF share: overall cancer HR 1.12 (95% CI 1.06–1.18); breast cancer HR 1.11 (1.02–1.22)	Single country; dietary data collected early in follow-up
Kliemann et al., 2023 (EPIC) [24]	Prospective cohort; 450,111 participants in 10 European countries	Intake (% g/day) by processing group; overall and site-specific incidence	Replacing 10% of processed foods with minimally processed foods lowered overall cancer risk; UPF intake showed the opposite gradient	Dietary instruments predate NOVA; substitution contrast specific to processed (Group 3) foods
Chang et al., 2023 (UK Biobank) [25]	Prospective cohort; 197,426 adults with repeated 24-h recalls	UPF as % of diet; overall and site-specific incidence and mortality	Higher UPF intake associated with higher overall cancer incidence and mortality; no association with colorectal cancer incidence	Self-selected subsample; short follow-up for rare cancer sites
Wang et al., 2022 (three US cohorts) [21]	Prospective cohorts; >200,000 US men and women	UPF servings per day; colorectal cancer incidence	Positive association in men (strongest for distal colon cancer); no significant association in women	Unexplained sex disparity; residual confounding
Zhong et al., 2023 (PLCO) [22]	Prospective cohort; US adults	UPF proportion of the diet; pancreatic cancer incidence	Higher intake associated with elevated risk; non-linear dose–response, robust to adjustment for BMI, diabetes, and diet quality	Single cohort; no independent replication
Li et al., 2024 [23]	Prospective cohort; 101,668 US adults	UPF intake; renal cell carcinoma incidence and RCC-specific mortality	Linear dose–response for both incidence and mortality; stronger associations below age 65	Single cohort; single exposure assessment
Lian et al., 2023 [26]	Systematic review and meta-analysis; 4 cohorts and 9 case–control studies	Highest vs. lowest UPF consumption category	Colorectal cancer risk approximately 31% higher in the highest category; positive association for breast cancer	Mixed study designs; heterogeneous UPF metrics
Meine et al., 2024 [53]	Systematic review and meta-analysis of gastrointestinal cancers	Highest vs. lowest UPF consumption category	Elevated colorectal cancer risk with a dose–response gradient	Heterogeneity across included studies
Bao et al., 2026 [51]	Umbrella review; 7 meta-analyses and 1 systematic review	Various UPF metrics; multiple cancer sites	Consistent positive direction for overall and colorectal cancer; certainty of evidence rated low to very low	Inherits limitations of primary studies
Jiang et al., 2026 [52]	Systematic review and dose–response meta-analysis; digestive system cancers	Various UPF metrics; digestive system cancer incidence	Positive associations overall; estimates stronger in case–control than in prospective cohort studies	Limited certainty; design-dependent estimates

## Data Availability

No new data were created or analyzed in this study. Data sharing is not applicable to this article.
